# Antenatal coffee and tea consumption and the effect on birth outcome and hypertensive pregnancy disorders

**DOI:** 10.1371/journal.pone.0177619

**Published:** 2017-05-16

**Authors:** Timothy van der Hoeven, Joyce L. Browne, Cuno S. P. M. Uiterwaal, Cornelis K. van der Ent, Diederick E. Grobbee, Geertje W. Dalmeijer

**Affiliations:** 1 Julius Center for Health Sciences and Primary Care, University Medical Centre Utrecht, Utrecht, The Netherlands; 2 Department of Pediatric Pulmonology, Wilhelmina Children’s Hospital, University Medical Centre Utrecht, Utrecht, The Netherlands; University of Tennessee Health Science Center, UNITED STATES

## Abstract

**Background and objective:**

Coffee and tea are commonly consumed during pregnancy. While several of their components, like caffeine, have strong pharmacological effects, the effect on the unborn fetus remains unclear. Caffeine intake has been associated with abortion, preterm birth and fetal growth restriction, but a general consensus on caffeine restriction is still lacking. We aimed to investigate antenatal coffee, tea and caffeine consumption and the effect on birth weight and length, gestational age at birth and hypertensive disorders in pregnancy.

**Methods:**

A total of 936 healthy pregnancies from the WHISTLER birth cohort with data on coffee and tea consumption were included. Maternal and child characteristics as well as antenatal coffee and tea consumption were obtained through postpartum questionnaires. Reported consumption was validated using available preconceptional data. Caffeine intake was calculated from coffee and tea consumption. Linear and logistic regression was used to assess the association with birth outcome and hypertensive disorders.

**Results:**

After adjustment for smoking and maternal age, a daily consumption of more than 300mg of caffeine compared to less than 100mg of caffeine was significantly associated with an increased gestational age (linear regression coefficient = 2.00 days, 95%CI = 0.12–4.21, P = 0.03). Tea consumption was significantly related to a higher risk of pregnancy induced hypertension (OR = 1.13, 95%CI = 1.04–1.23, P = 0.004). No associations concerning coffee consumption or birth weight and birth length were observed.

**Conclusions:**

Daily caffeine consumption of more than 300mg is possibly associated with an increase in gestational age at birth. A possible relation between high tea consumption and increased risk for pregnancy induced hypertension warrants further research. For most outcomes, we found no significant associations with coffee or tea intake.

## Introduction

Pregnancy is a delicate equilibrium between the mother and her unborn fetus. While fully dependent on mother’s blood supply, chemicals that freely pass the placenta can pose a risk for the developing child. There is increasing evidence that maternal lifestyle influences fetal development and can increase the risk for pregnancy complications and disease later in life.[1–3] Several of these lifestyle factors, such as smoking, drinking and obesity, have been identified, but uncertainty remains on the subject of nutrition. Some nutritional components, like folic acid, have been shown to contribute to a healthy pregnancy[4], while excessive intake of other components, like vitamin A, have proven harmful to the fetus.[5]

Coffee and tea are available worldwide and are commonly consumed during pregnancy.[6] While antenatal consumption of these beverages is generally socially accepted and deemed innocent, several of their components possess a strong pharmacological effect on the human body.[7,8] Caffeine is the major active chemical present in both coffee and tea and passes the placenta freely where the immature fetal liver enzyme CYP3A4 is unable to metabolize it.[9] Caffeine has been shown to increase epinephrine concentrations in mother and fetus resulting in decreased placental blood flow and hypoxia.[7,10]

Despite the known biological mechanisms of caffeine, clinical research does not observe a clear association with adverse pregnancy outcomes in population studies. Studies monitoring caffeine intake during pregnancy reported an increased risk of spontaneous abortion[11,12], fetal growth restriction[13–18] and preterm delivery[19,20]but an equal number of studies found no association.[20–27] Recently, two meta-analysis reported a higher risk of delivering low birth weight infants, while no association with preterm delivery was found.[28,29] These different results might be partly explained by the heterogeneity in study design, caffeine intake measurement and identification of confounders. Based on these results, caffeine restriction during pregnancy is encouraged in many national guidelines, but the large variety in cutoff values (0-300mg caffeine) underlines the inconsistency of current evidence.[30–34]

Some studies suggest a possible effect of chemicals present in coffee and tea other than caffeine, for instance polyphenols, which contribute to adverse pregnancy outcomes including preterm delivery and pre-eclampsia.[8,14,18,35] Therefore, studies investigating the effect of caffeine tend to overlook the fact that other components in coffee and tea may contribute. Finally, several studies suggest that antenatal coffee and tea consumption, besides adverse birth outcomes, can lead to hypertensive pregnancy disorders like pregnancy induced hypertension (PIH), (pre-)eclampsia or HELLP syndrome, but research on this subject is scarce.[8,36,37]

In this cohort study among 936 healthy Dutch pregnant women we aimed to investigate coffee, tea and caffeine intake during pregnancy and the effect on birth weight and length, gestational age at birth and hypertensive disorders in pregnancy.

## Materials and methods

### Study population

The dataset is part of the Wheezing-Illness-Study-LEidsche-Rijn (WHISTLER) study, an ongoing population-based birth cohort.[38] This study was initiated in 2001 and includes over 2500 healthy newborns from Leidsche Rijn, a new residential area near Utrecht, The Netherlands. Exclusion criteria were gestational age at birth less than 36 weeks, major congenital abnormalities and neonatal respiratory disease. When multiple children of the same mother were included, only the firstborn child was incorporated into the dataset.

Participants were recruited within 14 days after giving birth. The study was approved by the pediatric Medical Ethical Committee of the University Medical Center Utrecht. Written informed parental consent was obtained.

### Measurements

When offspring was four weeks of age, participants visited the clinic and information regarding ante-, peri- and postnatal factors was obtained through a questionnaire. These included maternal characteristics (age, height, weight, parity, ethnicity, educational level) and child characteristics (birth weight, birth length, sex, gestational age at birth, mode of delivery). Participants were asked about their average coffee, tea and alcohol consumption during pregnancy. Consumption was reported in cups per day, week, month or year. Coffee was specified as either caffeinated or decaffeinated. Due to later addition of the coffee and tea questions to the questionnaire, information about prenatal coffee or tea intake was available for only 936 of the 2500 participants, of which 887 reported information on both. Smoking was reported in number of cigarettes per day. Participants were asked if they suffered any specific disorders during pregnancy (hypertension, [pre-]eclampsia, diabetes, HELLP).

### Maternal variables

Coffee (caffeinated and decaffeinated) and tea intake was converted to cups/day. Caffeine intake was calculated from daily caffeinated coffee and tea intake. According to the Dutch Nutrition Centre caffeine content for one average serving (125mL) of coffee and tea is considered 85mg and 30mg, respectively.[32] Alcohol use in pregnancy was converted into IU/day. Smoking during pregnancy was converted into a total number of cigarettes smoked during pregnancy, taking duration of pregnancy and antenatal smoking cessation into account.

Ethnicity was based on western or non-western origin of the mother. Mothers were considered of western origin when born in Europe (excl Turkey), North America, Oceania, Indonesia or Japan.

### Birth outcome

Birth weight was assessed by calculating internal Z-scores, adjusted for gestational age at birth, birth length and sex. Birth length was assessed by calculating internal Z-scores, adjusted for gestational age at birth, birth weight and sex. Small for gestational age (SGA) was considered a weight below the 10^th^ percentile for gestational age at birth. SGA was calculated by comparing birth weight and gestational age at birth using reference charts from the Netherlands Perinatal Registry.[39]

### Data analysis

Linear regression was used to assess the relation between coffee, tea and caffeine consumption and continuous birth outcomes. Caffeine intake, tea intake and coffee intake were entered as independent variables in separate models. Birth weight, birth length and gestational age at birth were entered as dependent variables in separate linear models. Pregnancy hypertensive disorders and SGA were dichotomized and entered as dependent variables in separate binary logistic regression models. After univariable analysis, models were adjusted for smoking and age of mother as they were considered confounders. To assess a possible threshold effect, caffeine consumption was converted into categories and analyzed using dummy variables in linear regression. Since ethnicity data was available for 86.5% of the mothers, we checked in a separate model whether adjusting for ethnicity influenced the results. All statistical analyses were conducted using IBM SPSS (version 21 for Windows). Statistical significance was assumed for a two-sided P value <0.05.

## Results

### Population characteristics

936 mothers and their children were included in the analysis. Baseline characteristics are presented in Tables 1 and 2. Mothers had a mean age of 32.4 years (standard deviation [SD] 4.0 years). Between caffeine intake groups, maternal age was significantly higher in the high intake group, also ethnicity differed significantly (p<0.001 and p = 0.01 respectively). Of all mothers, 92.2% had any daily intake of caffeine (>0 mg). On average mothers consumed 177.6 mg (SD 124.7 mg) of caffeine per day, the majority of total caffeine (57.8%) being accounted for by tea. Alcohol use and smoking habits were low in our population, with a prevalence of 15.4% and 5.0%, respectively. No significant differences in alcohol consumption or smoking habits between caffeine intake groups were found.

**Table 1:** Baseline characteristics of mothers.

	Low caffeine intake <200mg/day		High caffeine intake ≥200mg/day		
	n = 540		n = 347		
Measure	Value^a^	n^b^	Value^a^	n^b^	p
**Age (years)**	32.0 ± 4.1	540	33.0 ± 3.7	347	<0.001
**Height (cm)**	170.0 ± 7.2	467	169.8 ± 13.2	306	0.74
**Weight (kg)**	71.5 ± 13.0	466	72.7 ± 12.8	301	0.18
**BMI (kg/m**^**2**^**)**	24.7 ± 3.8	464	24.8 ± 3.8	297	0.84
**Parity**		540		347	0.09
0	274 (50.7)		155 (44.7)		
>1	266 (49.3)		192 (55.3)		
**Ethnicity**		461		306	0.03
Western origin	399 (86.6)		281 (91.8)		
Other	62 (13.4)		25 (8.2)		
**Education**		460		300	0.92
Low (lower secondary school)	6 (1.3)		4 (1.3)		
Middle (upper secondary school)	118 (25.7)		73 (24.0)		
High (university)	336 (73.0)		223 (74.3)		
**Coffee consumption in pregnancy** cups/day	0 (0–1)	540	2 (1–2)	347	<0.001
**Tea consumption in pregnancy** cups/day	3 (1–4)	540	4 (2–7)	347	<0.001
**Caffeine intake in pregnancy** mg/day	115 (60–150)	540	265 (230–345)	347	<0.001
**Smoking in pregnancy** cig/week	34.8 (21–70)	14	35 (28–70)	15	0.15
**Alcohol consumption in pregnancy** units/week	0.06 (0.02–0.14)	70	0.06 (0.04–0.21)	70	0.09

^a^ Mean ± SD or number of cases (%) or Median (Q1-Q3).

^b^ Number of cases.

**Table 2:** Baseline characteristics of children.

	Low caffeine intake < 200mg/day		High caffeine intake ≥ 200mg/day		
	n = 540		n = 347		
Measure	Value^a^	n^b^	Value^a^	n^b^	p
**Gestational age at birth (weeks)**	39.8 ± 1.3	540	39.9 ± 1.3	347	0.15
**Sex**		540		347	0.86
Male	260 (48.1)		170 (49.0)		
**Weight (g)**	3524.2 ± 476.1	540	3582.3 ± 521.9	347	0.09
**Length (cm)**	50.7 ± 2.2	506	50.9 ± 2.2	316	0.36
**Mode of delivery**		536		343	0.17
Normal vaginal	396 (73.9)		258 (75.2)		
Vacuum/forceps	47 (8.8)		39 (11.4)		
C-section	93 (17.4)		46 (13.4)		
**Small for gestational age**	24 (4.4)	540	15 (4.3)	347	1.00

^a^ Mean ± SD or number of cases (%).

^b^ Number of cases.

### Coffee, tea, caffeine and birth outcome

Table 3 shows the associations between coffee, tea and total caffeine consumption and birth outcome. After adjustment for smoking habits and maternal age, higher maternal coffee intake was significantly associated with an increased birth weight with a regression coefficient of 31.26 g/cup (95%CI: 6.75–55.78, p = 0.02). This association lost statistical significance after calculating Z-scores adjusted for birth length, gestational age at birth and gender, although a positive tendency remained (linear regression coefficient 0.04 SD/cup, 95%CI: -0.02–0.09, P = 0.16). No statistically significant associations were observed between coffee, tea or caffeine consumption and birth length or SGA. A sensitivity analysis for ethnicity did not change the results.

**Table 3:** Coffee, tea, caffeine consumption and child outcome measurements.

Predictor	Unadjusted birth weight (g)	Z-score birth weight (SD)^a^	Z-score birth length (SD)^b^	Gestational age at birth (days)	Small for gestational age
	B (95% CI)	B (95% CI)	B (95% CI)	B (95% CI)	OR (95% CI)
**Coffee (cup/day)**	23.55 (0.11–47.00)*	0.03 (-0.02–0.08)	-0.03 (-0.07–0.02)	0.37 (-0.06–0.79)	0.95 (0.75–1.22)
Adjusted^c^	31.26 (6.75–55.78)*	0.04 (-0.02–0.09)	-0.02 (-0.07–0.03)	0.42 (-0.03–0.86)	0.93 (0.73–1.18)
**Tea (cup/day)**	8.13 (-4.49–20.75)	0.01 (-0.02–0.04)	0.003 (-0.03–0.02)	0.16 (-0.07–0.39)	0.92 (0.81–1.06)
Adjusted^c^	6.05 (-6.59–18.69)	0.01 (-0.02–0.03)	0.004 (-0.03–0.02)	0.15 (-0.08–0.38)	0.93 (0.81–1.07)
**Caffeine (100mg/day)**	15.32 (-19.85–41.48)	0.02 (-0.04–0.07)	-0.02 (-0.08–0.03)	0.41 (-0.07–0.88)	0.92 (0.70–1.21)
Adjusted^c^	17.89 (-8.67–44.46)	0.02 (-0.04–0.07)	-0.02 (-0.08–0.04)	0.43 (-0.06–0.91)	0.92 (0.71–1.19)

^a^ Birth weight adjusted for gestational age at birth, birth length and sex.

^b^ Birth length adjusted for gestational age at birth, birth weight and sex.

^c^ Adjusted for smoking and maternal age.

* P-value < 0.05.

There was a trend in association between intake of coffee, tea and caffeine and increased gestational age at birth (Caffeine: linear regression coefficient 0.43 day/100mg caffeine, 95%CI: -0.06–0.91, P = 0.09). Table 4 shows the threshold model with caffeine consumption converted into four categories. After adjustment a daily consumption of more than 300mg of caffeine compared to less than 100mg of caffeine was associated with a 2.00 days (95%CI: 0.07–3.93, P = 0.03) increased gestational age at birth. This association lost statistical significance after adjusting for ethnicity (Table 5), although a positive tendency remained (1.76 days, 95%CI: -0.29–3.81, P = 0.09).

**Table 4:** Threshold model^a^.

	n	Z-score birth weight (SD)^b^	Gestational age at birth (days)
Caffeine		B (95% CI)	B (95% CI)
< 100mg^c^	260	ref	ref
100-200mg	280	-0.03 (-0.20–0.15)	1.49 (-0.04–3.01)
200-300mg	220	0.04 (-0.14–0.23)	1.45 (-0.18–3.08)
≥300mg	127	0.09 (-0.13–0.32)	2.00 (0.07–3.93)*

^a^ Adjusted for smoking and maternal age.

^b^ Birth weight adjusted for gestational age at birth, birth length and sex.

^c^ Reference category.

* P-value < 0.05.

**Table 5:** Threshold model adjusted for ethnicity^a^.

	n	Z-score birth weight (SD)^b^	Gestational age at birth (days)
Caffeine		B (95% CI)	B (95% CI)
< 100mg^c^	214	ref	ref
100-200mg	247	-0.05 (-0.23–0.14)	1.31 (-0.32–2.95)
200-300mg	192	0.05 (-0.15–0.25)	1.08 (-0.66–2.83)
≥300mg	114	0.13 (-0.10–0.36)	1.76 (-0.29–3.81)

^a^ Adjusted for ethnicity, smoking and maternal age.

^b^ Birth weight adjusted for gestational age at birth, birth length and sex.

^c^ Reference category.

* P-value < 0.05.

### Coffee, tea, caffeine and hypertensive pregnancy disorders

Associations between coffee, tea and total caffeine consumption and hypertensive pregnancy disorders are shown in Table 6. While there were no statistically significant associations between coffee or caffeine and hypertensive pregnancy disorders, tea consumption was statistically significantly related to a higher risk of pregnancy induced hypertension (OR = 1.13, 95%CI: 1.04–1.23, P = 0.004). A sensitivity analysis for ethnicity did not change the results.

**Table 6:** Coffee, tea, caffeine consumption and hypertensive pregnancy disorders.

Predictor	Pregnancy induced hypertension	(Pre-)eclampsia	HELLP
	OR (95% CI)	OR (95% CI)	OR (95% CI)
**Coffee (cup/day)**	0.92 (0.75–1.12)	0.82 (0.47–1.43)	1.20 (0.76–1.89)
Adjusted^a^	0.94 (0.77–1.16)	0.81 (0.46–1.44)	1.30 (0.70–2.43)
**Tea (cup/day)**	1.13 (1.04–1.23)*	1.04 (0.83–1.30)	1.21 (0.89–1.63)
Adjusted^a^	1.13 (1.04–1.23)*	1.03 (0.83–1.29)	1.19 (0.88–1.61)
**Caffeine (100mg/day)**	1.05 (0.87–1.27)	0.99 (0.61–1.60)	1.44 (0.95–2.17)
Adjusted^a^	1.09 (0.89–1.33)	0.99 (0.59–1.67)	1.81 (0.92–3.54)

^a^ Adjusted for smoking and maternal age.

^*^ P-value < 0.05.

## Discussion

### Birth outcome

This study demonstrates that birth weight and birth length are not associated with coffee or tea consumption or caffeine intake during pregnancy. A daily caffeine consumption of more than 300mg is possibly associated with an increase in gestational age at birth. A similar tendency was observed for coffee, tea and caffeine consumption as continuous variables, but none of these associations were statistically significant.

The largest cohort study studying the effects of antenatal caffeine consumption and birth outcome (n = 59 123) Sengpiel et al (2013) in Norway, found total caffeine intake associated with decreased birth weight and higher risk for small for gestational age (SGA).[18] Similar to our findings, they reported an increased gestational age at birth of 8 hours per 100mg of caffeine a day, but only for caffeine from coffee. They attribute this outcome to a different compound present only in coffee and to differences in lifestyles between coffee and non-coffee drinkers. A consideration we would add to explain the association between coffee consumption and pregnancy is that women with a healthy pregnancy and a term baby may be less encouraged to reduce their caffeine intake. When hypertension or other health risks are present, doctors might discourage them from caffeine consumption, resulting in a higher coffee intake in the healthier women.

Our finding of a threshold effect with an intake of more than 300mg of caffeine a day is in agreement with findings from other studies and suggests that like many agents, caffeine may only be harmful in high doses.[16,40–42]

The only existing randomized controlled trial studied the effect of caffeine restriction and was performed by Bech et al (2007) in a Danish cohort of 1207 women.[21] After randomization women started drinking either caffeinated or decaffeinated coffee during the second half of pregnancy, but no effects were observed on either birth weight or gestational age at birth. However, only a difference in three cups of coffee (approximately 240mg of caffeine) was studied, while our study and others observe an effect above 300mg. Secondly, a possible effect of caffeine in the first half of pregnancy remained unexplored.

### Hypertensive disorders

We also found that tea consumption was associated with a statistically significant increase in the risk of pregnancy induced hypertension. A possible mechanism is through caffeine and polyphenol chlorogenic acid, both present in tea, that have been shown to increase homocysteine levels in humans.[43,44] Elevated homocysteine levels are associated with an increased risk of PIH and pre-eclampsia.[45] However, chlorogenic acid is also present in coffee, for which we found no association with PIH. Another potential mechanism is posed by Wei et al.[8] who found tea consumption significantly related to higher risk for pre-eclampsia. They ascribed this effect to chemicals present in tea other than caffeine, possibly flavonoids. Flavonoids, also a type of polyphenol, have been ascribed anti-oxidant effects, but a recent study showed an opposite effect with increased oxidative stress as a result. In the general population, the effect of tea on blood pressure remains controversial.[46]

Coffee and caffeine consumption were not associated with any hypertensive disorders, although with caffeine consumption a near significant increased risk for HELLP syndrome was observed. This observation could be due to chance since the prevalence of HELLP syndrome was very low in this cohort (n = 4, 0.4%).

Besides smoking habits, alcohol was considered a confounder, but due to the minimal alcohol consumption in this cohort, adjustment had a negligible effect on results and was omitted from the analysis.

### Limitations and strengths

**Fig 1:**
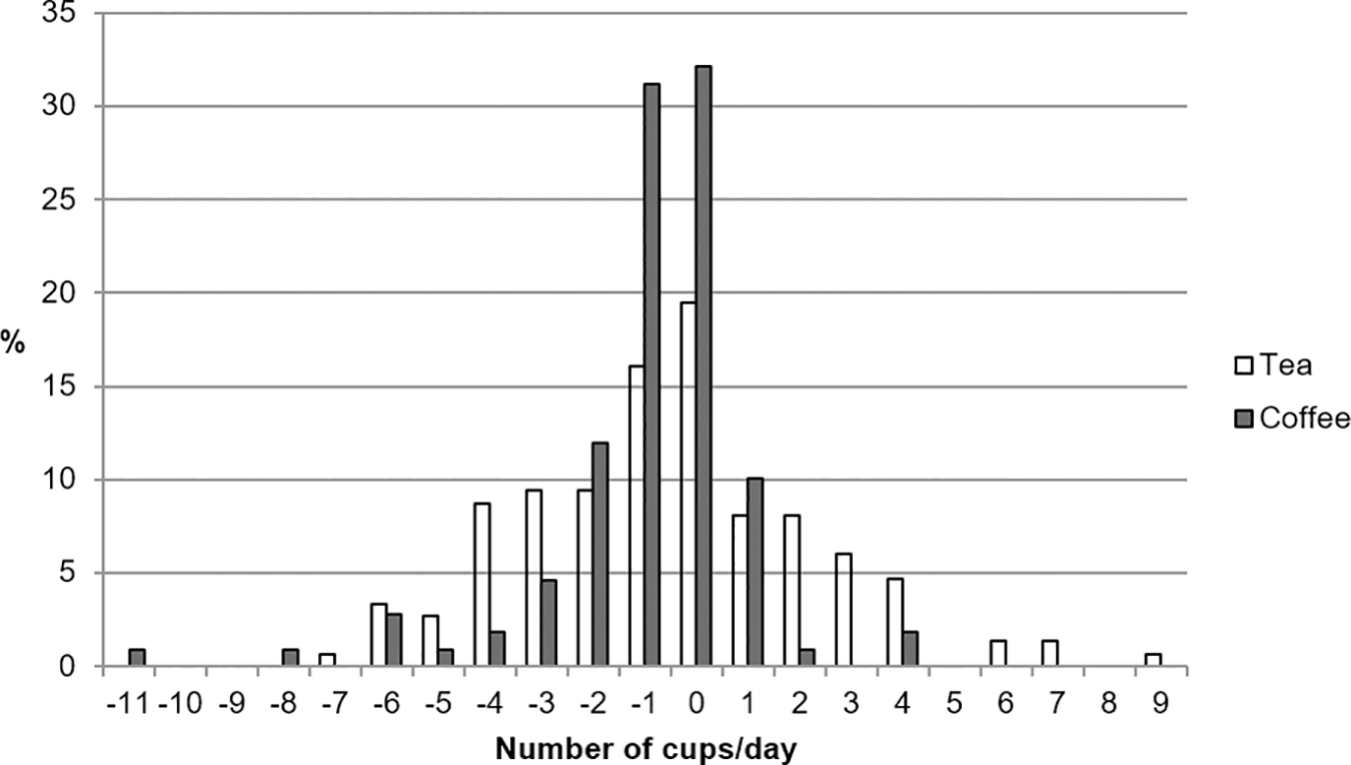
Intake difference—Preconceptional vs postpartum questionnaires.

Strengths of this study comprise the separate analysis of coffee, tea and caffeine intake and the inclusion of hypertensive pregnancy disorders as outcome in a healthy group of unselected women. The mean birth weight, birth length and incidence of hypertensive disorders in our sample are comparable to national reports[47,48], which means our sample properly reflects the general healthy Dutch population. The Netherlands is one of the five countries worldwide with the highest coffee consumption, reflecting a relatively large distribution of coffee consumption and contrasts in degree of exposure.[49] Another strength of this study is the availability of pre-pregnancy coffee consumption in a subset of participants, included to validate the reported consumption and mitigate the risk of postpartum questionnaires recall bias. For over a hundred participants data on tea and coffee consumption (149 and 109 participants, respectively) before conception was available.[50] Fig 1 shows a homogenous distribution of difference in drinking behavior, indicating most women did not change their intake in pregnancy, thus strengthening the accuracy of the data used in this study.

Some limitations have to be taken into account. Firstly, beverage consumption was aggregated for overall pregnancy and not available per trimester, while consumption may not be constant during trimesters by which effects of intake may vary. However, large studies that incorporated trimester specific questionnaires showed no difference between trimesters.[16,18] Secondly, this study was performed in a cohort of healthy pregnant women with uncomplicated singleton pregnancies and a minimum gestational age at birth of 36 weeks. Therefore, the findings can only be generalized to a healthy obstetric population with a low risk of complications. Thirdly, caffeine content for coffee and tea was estimated based on national nutritional values, because measurement of precise caffeine concentration for each serving was impossible. No differentiations were made between types of coffee or tea, except for decaffeinated coffee, which was considered to contain no caffeine.

Despite our efforts to validate the accuracy of the reported intake, the retrospective design of this study makes our data subject to recall bias. Next, for only 86.5% of the mothers ethnicity was known. Therefore we chose to do a sensitivity analyses to investigate the effect of ethnicity on our results. Also, we could not adjust for total energy, specific nutrient intake or caffeine intake from other sources like chocolate, cola or energy drinks, due to lack of data.

Finally, we cannot exclude the possibility of residual confounding.

Our findings suggest a possible effect of caffeine in prolonged gestational age at birth and an association between tea consumption and pregnancy induced hypertension. This considered, we cannot rule out that caffeine, or tea and coffee use in general, is harmful for the developing fetus. Dutch national guidelines recommend a caffeine restriction of 300mg or less but do not advise active preconceptional counseling on caffeine.[32,33] Other national guidelines recommend a restriction of either 200 or 300mg of caffeine a day during pregnancy. There is need for a large well-designed trial that addresses the inconclusiveness on this subject.

The association between tea and pregnancy induced hypertension has been scarcely studied, while biological evidence suggests a potential mechanism. Moreover, possible adverse health effects of tea, the second-most consumed beverage worldwide, will have substantial global impact. More research on this subject is needed.

In conclusion, a daily caffeine consumption of more than 300mg is possibly associated with a modest increase in gestational age at birth. There is no evidence that birth weight is associated with coffee or tea consumption or caffeine intake during pregnancy. A possible relation between high tea consumption and increased risk for pregnancy induced hypertension warrants further research.
